# CNS Infections Caused by Brown-Black Fungi

**DOI:** 10.3390/jof5030060

**Published:** 2019-07-10

**Authors:** Jon Velasco, Sanjay Revankar

**Affiliations:** 1 Detroit Medical Center, Wayne State University, Detroit, MI 48201, USA; 2 Division of Infectious Diseases, Department of Medicine, Wayne State University, 3990 John R. Street, 5 Hudson, Detroit, MI 48201, USA

**Keywords:** dematiaceous, brain abscess, fungal meningitis, voriconazole, posaconazole, *Cladophialophora*, Rhinocladiella, Exserohilum

## Abstract

Central nervous system (CNS) infections caused by brown-black or dematiaceous fungi are distinctly rare and represent a small proportion of infections termed phaeohyphomycoses. However, these are becoming more commonly reported. Though many fungi have been implicated in disease, most cases are caused by only a few species, *Cladophialophora bantiana* being the most common. Most of the fungi described are molds, and often cause infection in immunocompetent individuals, in contrast to infection with other more common molds such as *Aspergillus*, which is usually seen in highly immunocompromised patients. Diagnosis is challenging, as there are no specific tests for this group of fungi. In addition, these infections are often refractory to standard drug therapies, requiring an aggressive combined surgical and medical approach to improve outcomes, yet mortality remains high. There are no standardized treatments due to a lack of randomized clinical trials, though guidelines have been published based on available data and expert opinion.

## 1. Introduction

Central nervous system (CNS) infections caused by fungi are generally uncommon, and infections due to brown-black or dematiaceous fungi are distinctly rare. However, case reports are becoming more common and awareness of these infections is growing, in part due to widely publicized outbreaks of infection, often in association with contaminated therapeutics [1,2,3]. Most of the fungi described are molds, and often cause infection in immunocompetent individuals, in contrast to infection with other more common molds such as *Aspergillus*, which is usually seen in highly immunocompromised patients [1]. There is emerging evidence that specific genetic mutations may be associated with an increased risk for these infections in certain individuals [4,5]. While there have been significant advances in the diagnosis of more common fungal infections due to *Candida* and *Aspergillus*, there are no specific diagnostic tests for dematiaceous fungi, adding to the challenge of their management. In addition, these infections are often refractory to standard drug therapies, and often require combined surgical and medical therapies [6]. Since these are so infrequently seen in clinical practice, randomized clinical trials are impractical and there is no consensus regarding treatment. We will review the mycologic and clinical aspects of these infections and summarize the available data on management.

## 2. Mycology 

While over 150 species and 70 genera of brown-black fungi have been implicated in human disease, relatively few have been reported as a cause of CNS infection [1]. *Cladophialophora bantiana* is by far the most common species isolated, while other fungi such as *Rhinocladiella (*formerly *Ramichloridium) mackenziei, Verruconis (*formerly *Ochroconis) gallopavum*, and *Wangiella dermatitidis* are also frequently seen in this clinical setting [1]. A list of common species is presented in Table 1. A more recent mold that has been reported is *Fonsecaea monophora* [7,8]. The distinguishing characteristic common to all brown-black fungi is the presence of high amounts of melanin in their cell walls, which imparts the dark color to their conidia or spores and hyphae [9]. The colonies are typically brown to black in color as well. Dematiaceous fungi are generally found in soil or associated with plants and are distributed throughout the world. Environmental surveys of outdoor air for fungal spores often isolate these molds [10]. Occasionally, species appear to be geographically restricted, such as *R. mackenziei*, which has primarily been seen in patients from the Middle East [11]. Exposure is generally thought to be from inhalation or minor trauma, which may not even be noticed by the patient. Since these fungi are ubiquitous in the environment, individuals are constantly exposed to them, though they rarely cause disease. 

Guidelines are available regarding the handling of potentially infectious fungi in the laboratory setting [12]. Cultures of certain well-known pathogenic fungi, such as *Coccidioides immitis* and *Histoplasma capsulatum,* are suggested to be worked with in a Biosafety Level 3 facility, which requires a separate negative pressure room [12]. Recently certain agents of phaeohyphomycosis, in particular *Cladophialophora bantiana,* have been included in the list of fungi that should be kept under Biosafety Level 3 containment, even extending to special infection control measures for hospitalized patients [13,14]. This is based on their potential for causing life-threatening infection in normal individuals, though no laboratory or nosocomial transmission has been documented to date. 

## 3. Pathogenesis

Brown-black fungi frequently cause CNS disease in immunocompetent individuals, and the pathogenic mechanisms involved in this type of infection remain largely unknown and have not been well studied. Numerous in vitro and animal studies have shown that cell wall-associated melanin, found in all dematiaceous fungi, is likely a major virulence factor [15,16]. It is able to scavenge free radicals and hypochlorite that are produced by phagocytic cells in their oxidative burst that typically kills most organisms [15]. In addition, melanin appears to bind to hydrolytic enzymes, preventing their action on the plasma membrane [16]. These functions may help explain the pathogenic potential of some dematiaceous fungi, particularly in normal hosts. Melanin has also been found in non-pigmented fungi such as *Histoplasma capsulatum* and *Cryptococcus neoformans,* and shown to increase resistance to the activity of amphotericin B and caspofungin, possibly by binding these drugs [17]. A recent genomic analysis of *C. bantiana* revealed genes that may be involved in tolerating thermal stress, which may enhance its ability to cause systemic infection [18].

Recent studies have elucidated an intriguing association with certain genetic markers and an increased risk of fungal infection [5]. Mutations in the caspase activation and recruitment domain (CARD) protein, specifically CARD9, that cause a functional CARD9 deficiency have been hypothesized to impair the killing of fungi by mononuclear and/or microglial cells at the blood-brain barrier due to impaired production of certain inflammatory markers [19]. CARD9 was discovered in 2000 and the first case of deficiency was documented in 2009 [20]. It is only seen with autosomal recessive inheritance, with about 50 cases reported to date [20]. Murine models of CARD9 knockout mice have demonstrated that cytokine response is impaired in association with more severe disease due to *Exophiala* and *Phialophora* species [21,22]. A number of reports have documented patients with CARD9 deficiency that have disseminated infection due to various dematiaceous fungi, including *Exophiala*, *Corynespora*, and *Phialophora* [5,19,21,23]. An interesting observation regarding this association is that only single fungal, ascomycete species have caused infection in these patients, and no pulmonary infections have been observed, despite likely exposure to airborne molds [5,20]. Further research is needed to improve our understanding of this association.

## 4. Diagnosis

The diagnosis of CNS infections due to brown-black fungi requires a high degree of clinical suspicion, as these are rarely seen in practice. Careful assessment of signs and symptoms should lead to further imaging and/or acquisition of appropriate tissue samples for diagnostic testing. In the case of brain abscess, patients are initially often assumed to have a malignancy based on imaging, and only after pathologic examination reveals hyphae does the consideration of fungal infection arise. Radiologically, specific findings on magnetic resonance imaging (MRI) may suggest these unusual infections, with restricted diffusion and a ‘double ring sign’ observed in certain cases [24,25]. From tissue samples, a simple potassium hydroxide preparation from the lesion may show pigmented hyphae. In pathologic specimens, they are best observed with the Fontana-Masson stain, which is specific for melanin [9]. This is useful in differentiating these fungi from other more common molds, such as *Aspergillus*. In addition, hyphae typically appear more fragmented in tissue than seen with *Aspergillus*, with irregular septate hyphae and yeast-like forms [1]. However, it may still be difficult to differentiate this pathologically from infection due to other molds. 

Unlike for more common fungi that cause human disease, there are no specific serologic, antigen or polymerase chain reaction (PCR) tests readily available to detect dematiaceous fungi in blood or tissue, which is primarily due to the incredible diversity of these pathogens. However, antigen tests that have been primarily useful for *Aspergillus* and *Candida*, such as serum or bronchoalveolar lavage galactomannan and serum β-D-glucan (BDG), occasionally may be positive in infections with dematiaceous fungi, but this is not consistent [26,27,28]. Unfortunately, no commercially available diagnostic tests are available to identify these fungi to the species level.

Studies have begun to examine the potential of identifying species within this diverse group of fungi using PCR of highly conserved regions of ribosomal DNA [1]. Different components of fungal rRNA, namely, the internal transcribed spacer (ITS) region, have been used to provide reliable species identification and differentiation between strains, though this is labor intensive and time consuming [29,30]. Molecular identification has become more useful with studies identifying clinical isolates of dematiaceous fungi to the genus or species level by sequencing a combination of the ITS region and the D1/D2 variable region of 28S rDNA [31,32]. More rapid methods are being developed such as amplified restriction length polymorphism analysis and rolling circle amplification of ITS sequences that may have greater clinical utility [33,34].

Matrix Assisted Laser Desorption Ionization Time of Flight (MALDI-ToF) mass spectrometry is a useful method for microbial identification [35]. It is broadly effective across different groups of organisms grown in different conditions. It is also rapid, providing results within a few minutes, though samples from an actively growing culture are needed. Sensitivity is good, needing only 10^4^ organisms for detection [35]. However, difficulties in identifying molds arise due to differences in spores vs. hyphae and the inherent biological complexity of fungal organisms. Clinically important fungi have been included in most databases, though uncommon molds are generally not reliably identified [28,36,37]. Further database refinement is needed in order to be clinically useful, though this is time consuming and laborious. MALDI-ToF for dematiaceous fungal identification is still a work in progress [28,37]. 

Currently, the diagnosis of these infections initially relies on pathologic examination of clinical specimens and careful gross and microscopic examination of cultures, occasionally requiring the expertise of a mycology reference lab for unusual or newly described pathogens. Despite the various testing methodologies available in the modern clinical laboratory, culture remains the mainstay of diagnosis. 

## 5. Clinical Presentation and Management

Central nervous system infection caused by brown-black fungi most frequently presents as primary brain abscess in immunocompetent individuals, and is one of the most unusual manifestations of phaeohyphomycosis. A review of 101 cases of CNS infection due to dematiaceous fungi found 87 cases of brain abscess, and 9 cases of meningitis [38]. Interestingly, the majority of cases occurred in patients with no risk factor or immunodeficiency. The predilection of these fungi to cause CNS disease in immunocompetent individuals is remarkable and unique among fungi. Typical symptoms included headache, neurologic deficits, and seizures, though fever was uncommon. Clinical management in this case series varied, though combination antifungal therapy appeared to be more effective than monotherapy and complete excision of brain lesions more effective than simple aspiration. Subsequent case studies have had similar findings, though specific antifungal regimens that optimize clinical outcomes remain elusive [39,40]. Other cases with myelitis have been reported, usually due to *C. bantiana* [41,42]. Mortality rates are high, regardless of underlying immune status or clinical approach.

### 5.1. Brain Abscess

This is the most common manifestation of CNS infection, and many patients have no apparent immunodeficiency [43,44,45,46]. However, among immunocompromised patients there are varying degrees of immune suppression, including chemotherapy, steroids, and organ and stem cell transplantation [6,47,48,49]. Rarely, altered mental status such as lethargy or personality changes have been observed [43,46]. Most patients have a subacute presentation, though some noted symptoms for only a few days and others with neurologic findings for months before being diagnosed [43,46]. There is usually a single lesion seen on brain imaging, though multiple abscesses can occur, with resulting poor prognosis due to inability to completely resect all lesions [47,49]. Other potential risk factors have also been noted in the literature, including head trauma, concurrent sinusitis, and even smoking marijuana [50,51,52]. Cerebrospinal fluid analysis, when safe and indicated, may be normal [53]. 

Mold-active triazoles are the mainstay of therapy due to generally broad activity against dematiaceous fungi [54,55]. Voriconazole has become the most commonly used agent, due to its broad spectrum of activity against dematiaceous fungi and good CSF penetration [6,27]. Posaconazole is a useful alternative, with excellent in vitro activity, tolerability and increasing clinical experience in these infections [56,57]. Notably, it has also been used successfully in cases of *Rhinocladiella* brain abscess, which is almost uniformly fatal [58,59]. Isavuconazole, a more recent arrival, is also well tolerated with good in vitro activity, though minimal clinical experience to date [6]. Itraconazole has had extensive clinical experience, though relatively poor tolerability and an FDA black box label has relegated it to salvage therapy except in resource poor regions [6]. Amphotericin B does not have consistent activity against these fungi, though use of lipid formulations of amphotericin B may allow for better efficacy by allowing use of much higher doses than possible with standard amphotericin B and likely improved CNS penetration. *C. bantiana* has a unique susceptibility to flucytosine, which has shown efficacy in combination with other agents in animal models and case series [38,60]. However, monotherapy with any of these agents is generally ineffective, leading many clinicians to use combination antifungal therapy, which is complicated since there are no standardized regimens. Murine models involving *C. bantiana* and *Exophiala* suggest that posaconazole may have the best activity as a single agent, and combinations using posaconazole have also been found to be more effective [61,62,63,64]. In one study, the combination of posaconazole, micafungin and flucytosine was most effective in a murine model of *C. bantiana* brain infection [61]. While compelling, animal models are limited in predicting which therapies are actually associated with clinical response. A retrospective analysis of cases indicated that the combination of amphotericin B, flucytosine, and itraconazole was associated with improved survival, though this was based on a small number of patients [38]. Guidelines from the European Society of Clinical Microbiology and Infectious Diseases suggest voriconazole, flucytosine and an echinocandin, with or without amphotericin B, based on animal models and case reports [65]. In addition, multiple retrospective studies indicate that complete excision of brain abscesses appears to improve outcomes [38,39,40]. There are reports of complete excision alone leading to apparent cure [66]. Despite these measures, overall mortality is often over 70% [38]. In light of this, some patients, especially those receiving immunosuppressive drugs, are placed on indefinite maintenance therapy with voriconazole or posaconazole once the infection has been successfully treated [57]. Given the difficulties in treating these patients, alternative management strategies have been attempted, similar to treatment of coccidioidal meningitis with intrathecal amphotericin B [67]. Since intravenous amphotericin B is often not effective, administration directly into the spinal fluid by the intrathecal route or even intracavitary instillation has been used in certain cases [44,45,68]. Clinical response was achieved in some of these cases, though other systemic therapy was also given, including voriconazole. Much work remains to be done to improve the management of these refractory infections.

### 5.2. Fungal Meningitis Outbreak

Meningitis is a very uncommon manifestation of infection of dematiaceous fungi. It may occur as an isolated clinical presentation or in association with brain abscess or encephalitis. There are few cases of naturally occurring infection, and outcomes reported are generally poor [38]. However, in 2012, a large outbreak of fungal meningitis occurred in the U.S. due to the mold *Exserohilum rostratum* [2]. It is a rare cause of phaeohyphomycosis, and prior to the outbreak, had never been associated with CNS disease [69]. It characteristically produces large conidia with a distinct, protruding hilum and darkly pigmented septum at both ends [70]. Skin, corneal, or sinus disease is common. Infections are usually seen in regions with tropical climates, such as India, Israel, and the southern U.S. [69]. Most patients with invasive and skin infections are immunocompromised, whereas keratitis and allergic fungal sinusitis are associated with local trauma and atopy, respectively [69]. *E. rostratum* was the predominant pathogen in the 2012-2013 multistate fungal meningitis outbreak associated with contaminated injectable methylprednisolone acetate products traced to the New England Compounding Center in Massachusetts [71]. During this outbreak, almost 14,000 patients were potentially exposed, with 89% occurring through epidural, spinal, or paraspinal injections and 12% through peripheral joint or other injections [71]. There were 753 reported cases of infection and 64 deaths (8%) [71]. Laboratory evidence of *E. rostratum* was present in specimens from 153 patients (20%) [2].

In a large series, clinical data were available for 728 of the patients, revealing that 43% had spinal or paraspinal infections only (epidural abscess, vertebral osteomyelitis, diskitis, arachnoiditis, and cauda equina syndrome), 31% had meningitis only, 20% had meningitis and concurrent spinal or paraspinal infections, 4% had septic arthritis after a peripheral-joint injection, 1% met the case definition for stroke due to presumed meningitis, and less than 1% had spinal or paraspinal infections and concurrent peripheral-joint infections [2]. The Centers for Disease Control (CDC, Atlanta, GA, USA) received reports of stroke in 40 case patients (5%), the majority of which were ischemic and involved the posterior circulation [2]. The median age of these patients was 64 years (range, 15 to 97), 59% of which were women. Only 8% of patients had underlying immunosuppression. Symptoms varied depending on the site of infection. Headache (88%) and neck stiffness (49%) were the most common symptoms with fever present in only 31% among patients with meningitis. In patients with spinal or paraspinal infections, back pain (63%) and headache (36%) were the most commonly reported symptoms. Joint pain was reported in 84% of patients with peripheral joint infections. The incubation period varied for each syndrome; the shortest incubation period was among patients with stroke without documented meningitis (i.e., in whom a lumbar puncture was not performed) with a median of 24 days (range, 3 to 157) and longest among patients with peripheral joint infections with a median of 65 days (range, 22 to 190). In patients who developed meningitis, the median leukocyte count in the initial cerebrospinal fluid sample was 83 cells per cubic millimeter (range, 6 to 15,400), the median glucose concentration was 53 mg/dL (range, 0.2 to 13.8), and the median protein level was 84 mg/dL (range, 13 to 2830) [2,71].

There were no established diagnostic standards at the beginning of the outbreak. Fluid and tissue cultures as well as histopathology were utilized to confirm the diagnosis. When the outbreak was recognized, PCR to detect fungal DNA using broad range primers was performed on samples from case-patients sent to the CDC. *E. rostratum* was identified as the predominant etiologic agent from the patient samples which led to the development of the *Exserohilum*-specific PCR primers, allowing the detection of an additional 22% of positive samples which were negative with the broad-range PCR [72]. The overall sensitivity of the fungal PCR was 29% compared to 14% sensitivity of culture. However, using both methods, the sensitivity improved to 33%. The PCR specificity was 100% [72]. Since many exposed patients had no new symptoms, screening MRI of the spine was performed on 172 of exposed patients who had not presented for medical care related to adverse effects after spinal or paraspinal injections [73]. MRI was abnormal in 36 (21%), showing epidural or paraspinal abscess or phlegmon, arachnoiditis, spinal osteomyelitis or diskitis, or moderate to severe epidural, paraspinal, or intradural enhancement which led to subsequent treatment [73]. 

There were few clinical data which existed to guide specific recommendations in the treatment of *E. rostratum* infections prior to the outbreak, and no experience treating meningitis. Intravenous amphotericin B and intravenous voriconazole (either alone or in combination) have been demonstrated to successfully treat invasive *E. rostratum* infections [69]. CDC treatment guidelines initially recommended high-dose dual therapy with voriconazole (6 mg/kg every 12 h) and liposomal amphotericin B (5–6mg/kg daily) [74]. The recommendations were later updated to voriconazole monotherapy for patients without serious illness. A minimum of 6 months of therapy was recommended for patients with severe disease and 3 to 6 months for less severe infections, though some patients required much longer courses of therapy [74,75]. Cerebrospinal fluid BDG testing (Fungitell, Associates of Cape Cod Inc., East Falmouth, MA) was found to be useful in monitoring treatment response with antifungals [76,77]. BDG concentrations in CSF < 138 pg/mL were considered “low” (baseline), BDG concentrations between 138 and 230 pg/mL were considered “indeterminate,” and had a sensitivity of 100% (95% CI, 95%–100%) and a specificity of 98% (95% CI, 83%–99%), and a BDG level >230 was considered “high” with a sensitivity of 99% (95% CI, 92%–100%) and the same specificity of 98% (95% CI, 81%–99%) [77]. It was hypothesized that the methylprednisolone itself may have contributed to the virulence of the infection, though in vitro studies did not confirm this [78,79]. Despite the magnitude of potentially exposed patients, less than 10% developed disease and fewer than 10% died even in the setting of direct inoculation into the CNS, although individual centers had higher infection rates [74,75]. A number of patients developed significant adverse effects that were likely related to prolonged use of high dose voriconazole, requiring adjustments to therapy [74,75].

## 6. Summary

Brown-black fungi are commonly encountered in the environment, though rarely cause disease in humans. They can be associated with a variety of infectious syndromes, and CNS disease is among the least common manifestation, though it has a unique predilection to occur in immunocompetent individuals. Brain abscess is the most frequent clinical manifestation, usually with a subacute onset. Biopsy for culture and histopathology is the most reliable means of diagnosis, though molecular methods are becoming more useful. Therapy is evolving, based on animal models, clinical experience and expert opinion. A typical approach is to attempt complete resection when feasible, followed by a combination of antifungal agents, depending on the fungal species involved. Duration of therapy is usually prolonged, with some clinicians pursuing indefinite oral azole therapy in selected cases, often in immunocompromised patients. The fungal meningitis outbreak demonstrated how little is known about many uncommon species, including optimal management of invasive infections. The attention focused on this tragedy will hopefully spur additional, much needed research into these rare but intriguing molds.

## Figures and Tables

**Table 1:** Fungal species causing CNS disease.

Fungal Species
*Cladophialophora bantiana* *Rhinocladiella mackenziei* *Lomentospora prolificans* *Verruconis gallopavum* *Chaetomium strumarium*
*Exophiala dermatitidis* *Fonsecaea monophora * *Bipolaris spicifera* *Curvularia lunata*
